# NF-ΚB Activation as a Key Driver in Chronic Lymphocytic Leukemia Evolution to Richter’s Syndrome: Unraveling the Influence of Immune Microenvironment Dynamics

**DOI:** 10.3390/genes15111434

**Published:** 2024-11-05

**Authors:** Paulo Rohan, Renata Binato, Eliana Abdelhay

**Affiliations:** Stem Cell Laboratory, Specialized Laboratories Division, Instituto Nacional de Câncer (INCA), Rio de Janeiro 20230-130, RJ, Brazil; rohanphn@gmail.com (P.R.); renata.binato@inca.gov.br (R.B.)

**Keywords:** chronic lymphocytic leukemia, Richter’s syndrome, NFKB, tumor microenvironment

## Abstract

**Background/Objectives**: Chronic lymphocytic leukemia (CLL) is the most common adult leukemia in Western countries and it can progress to Richter’s syndrome (RS), a more aggressive condition. The NF-κB pathway is pivotal in CLL pathogenesis, driven mainly by B-cell receptor (BCR) signaling. However, recent evidence indicates that BCR signaling is reduced in RS, raising questions about whether and how NF-κB activity is maintained in RS. This study aims to elucidate the triggers and dynamics of NF-κB activation and the progression from CLL to RS. **Methods**: Integrated single-cell RNA sequencing data from peripheral blood samples of four CLL–RS patients were analyzed. NF-κB pathway activity and gene expression profiles were assessed to determine changes in NF-κB components and their targets. Tumor microenvironment composition and cell–cell communication patterns were inferred to explore NF-κB regulatory mechanisms. **Results**: RS samples showed increased proportions of malignant cells expressing NF-κB components, including *NFKB1*, *NFKB2*, *RELA*, *IKBKG*, *MAP3K14*, *CHUK*, and *IKBKB*, with significantly higher expression levels than in CLL. Enhanced NF-κB pathway activity in RS cells was associated with targets involved in immune modulation. The tumor microenvironment in RS displayed significant compositional changes, and signaling inference revealed enhanced cell–cell communication via BAFF and APRIL pathways, involving interactions with receptors such as BAFF-R and TACI on RS cells. **Conclusions**: The findings from this study reveal an active state of NF-κB in RS and suggest that this state plays a critical role in the evolution of CLL to RS, which is modulated by alternative signaling pathways and the influence of the tumor microenvironment.

## 1. Introduction

Chronic lymphocytic leukemia (CLL) is an indolent malignancy characterized by the expansion and accumulation of mature clonal B lymphocytes in the peripheral blood (PB), bone marrow (BM), and secondary lymphoid organs (SLOs) [1]. CLL accounts for approximately 25–40% of all adult leukemias in Western countries [2,3]. From a molecular perspective, CLL can be divided into two main subtypes depending on the mutational status of the immunoglobulin heavy-chain variable region gene (IGHV): IGHV-mutated (IGHV-M) and IGHV-unmutated (IGHV-UM) CLL, each exhibiting distinct genetic profiles and prognostic outcomes [4]. Despite its generally indolent phenotype, in 2–7% of cases, CLL can progress to a more aggressive clinical form known as Richter’s syndrome (RS), which predominantly presents with diffuse large B-cell lymphoma (DLBCL) histology [5]. This transformation is associated with a poor prognosis, with a median survival of 1–5 years [6,7]. Furthermore, the molecular events contributing to this transformation have not been fully elucidated and require further investigation [8,9]. Among the various molecular pathways implicated in CLL progression, the nuclear factor kappa B (NF-κB) signaling pathway has emerged as a critical player.

The nuclear factor kappa B (NF-κB) family comprises five subunits—RelA (p65), RelB, c-Rel, NF-κB1 (p50/p105), and NF-κB2 (p52/p100)—which form homodimers or heterodimers to regulate a wide array of genes involved in immune responses, cell proliferation, apoptosis, and inflammation [10]. NF-κB activation occurs through two main pathways, the canonical and noncanonical pathways, each of which are regulated by distinct signaling mechanisms [11]. In B lymphocytes, NF-κB signaling plays a key role in development, differentiation, and survival [12]. The canonical pathway is primarily activated by B-cell receptor (BCR) engagement and TNFα, whereas the noncanonical pathway is induced by receptors such as the B-cell activating factor receptor (BAFF-R), B-cell maturation antigen (BCMA), transmembrane activator and CAML interactor (TACI), and CD40 [13,14].

In the context of CLL, constitutive activation of NF-κB has been identified as a central feature contributing to the survival and proliferation of leukemic cells [15,16]. Several genetic alterations affecting components of the NF-κB pathway have been described in CLL, and persistent BCR signaling provides continuous activation cues for this pathway [17,18,19,20]. However, the specific role of NF-κB activation in the evolution from CLL to RS is still poorly understood. Notably, recent studies have reported that RS malignant cells exhibit reduced BCR signaling compared with CLL cells, suggesting that alternative mechanisms may sustain NF-κB activity in RS [21]. Therefore, it is crucial to clarify whether and how NF-κB activity is modulated in RS, as well as the consequences of this modulation on disease biology and progression.

To address this knowledge gap, bioinformatics analyses were performed on single-cell RNA sequencing (scRNA-seq) data from paired CLL and RS samples to investigate the dynamics of NF-κB expression and modulation during disease progression. By integrating samples from different studies, changes in the composition of the immune microenvironment, cell–cell signaling, and differential target regulation were examined systematically. Not only increased expression of key members of the NF-κB pathway in RS, but also an increased percentage of cells expressing these members and increased activity of this pathway were identified. Moreover, it was demonstrated that distinct targets regulated by NF-κB in RS are involved in key processes such as immune system activation, cytokine production, B-cell proliferation, and amplification of the NF-κB signal itself. Concurrently, changes in the composition of the immune microenvironment along with alterations in the signaling patterns between immune cells and tumor cells were also found. This shift in signaling patterns indicated greater modulation of NF-κB activation through alternative pathways beyond the B-cell receptor (BCR), such as via BAFF and TACI.

When considered together, these findings provide insights into the activation state and regulatory mechanisms of NF-κB pathway dynamics in RS, as well as the consequences of these changes. These results not only shed light on the evolution from CLL to RS but also highlight possible mechanisms involved in this transformation.

## 2. Materials and Methods

### 2.1. Data Acquisition and Patient Selection

**Figure 1:**
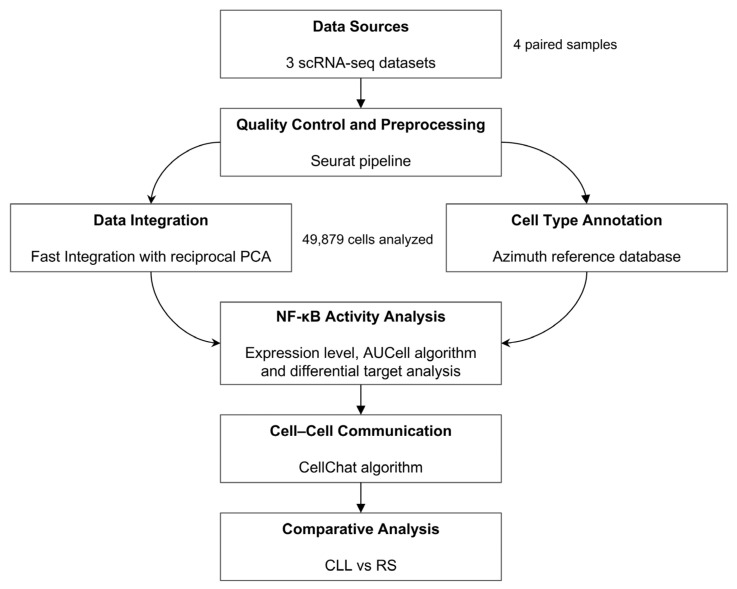
Single-cell RNA sequencing (scRNA-seq) and clinical data from three datasets were obtained from the GEO database. After patient selection, a total of four paired CLL–RS peripheral blood samples were selected. Quality control and preprocessing steps were then performed to eliminate low-quality cells. The samples were integrated via the RPCA method, resulting in a combined dataset of 49,879 cells. The cell types were annotated from the Azimuth database using a reference-based approach. Using the integrated dataset, NF-κB activity analysis steps, which addressed expression levels, pathway activity, and differential target analysis, were performed. Additionally, the annotated cell types were used to construct the cell–cell communication network, and comparative analyses between the CLL and RS conditions were subsequently performed to identify differences in signaling patterns.

scRNA-seq data from patients with paired peripheral blood samples from CLL and RS, as well as related clinical data, were obtained from the Gene Expression Omnibus (GEO) database (accession numbers: GSE165087 and GSE183432) via the GEOquery package (version 2.72.0) (GEOquery, RRID:SCR_000146) from R/Bioconductor (version 3.20, http://www.bioconductor.org/, accessed on 3 October 2024 ) (Bioconductor, RRID:SCR_006442) in R software (version 4.4.1, https://www.r-project.org/, accessed on 17 September 2024) (R software, RRID:SCR_001905) and the Zenodo repository (https://zenodo.org/, accessed on 6 September 2024, accession code: 6631966) [22]. After that, only patients who had paired samples of CLL and RS and did not have the IGLV3–21^R110^ mutation, which triggers autonomous BCR signaling and could confound the current analysis [23], were selected. This resulted in a sample set of four CLL–RS pairs: one pair from the GSE165087 study (samples GSM5025885 and GSM5025886), two pairs from the GSE183432 study (samples GSM6217976, GSM6217978, GSM6217981, and GSM6217983), and one pair from Zenodo-6631966 (patient 12, samples T1 and T6). The design and workflow of this study are shown in Figure 1.

### 2.2. Preprocessing and Dataset Integration

Each scRNA-seq sample was individually preprocessed to filter out low-quality cells via the Seurat package (version 5.0.1) (Seurat, RRID:SCR_016341) [24]. After visual inspection, the criteria used to consider cells of low quality were a percentage of mitochondrial genes greater than 20% (indicative of potential cell stress or apoptosis), number of detected genes less than 250, and number of identified unique molecular identifiers (UMIs) less than 900. The DoubletFinder package (version 2.0.4) (DoubletFinder, RRID:SCR_018771) was used to predict and eliminate doublets [25]. After that, the data were normalized, and variable features were obtained through the SCTransform (SCT) method, with dimensionality reduction through principal component analysis (PCA) [26]. The data from the different samples were then integrated via fast integration by the reciprocal PCA (RPCA) method, which effectively corrects for batch effects across datasets [24].

### 2.3. Cluster and Cell-Type Annotation

After data integration, clusters with a resolution of 0.5 were identified, which provided a balance between cluster granularity and interpretability. Both uniform manifold approximation and projection (UMAP) and t-distributed stochastic neighbor embedding (t-SNE) methods were used for dimensionality reduction and cluster visualization. Additionally, a reference-based annotation with the Azimuth database and the peripheral blood mononuclear cell (PBMC) dataset [27,28] was used. Manual curation was performed among the identified clusters and combined with automatic annotation to achieve high-confidence cell labels. Gene expression markers for the identified labels were obtained and used to refine and confirm the final cell-type annotations.

### 2.4. Expression Analysis of NF-κB Components and Pathway Activity

To verify the expression status of NF-κB components within and between groups, only the malignant cells were filtered, and then a two-pronged approach was adopted: the expression levels and the percentage of cells expressing each component were assessed using the scCustomize package (version 2.1.2) (scCustomize, RRID:SCR_024675). The components analyzed were *NFKB1* (p50/p105), *NFKB2* (p52/p100), *RELA* (p65), *RELB* (RelB), *REL* (c-REL), *IKBKG* (NEMO), *MAP3K14* (NIK), *CHUK* (IKK-α), and *IKBKB* (IKK-β) [10]. Additionally, a gene set of high-confidence NF-κB targets was obtained from the Collection of Transcriptional Regulatory Interactions (CollecTRI) via the decoupleR package (version 2.10.0) [29,30]. This gene set was used to calculate the activity level of the NF-κB pathway in each individual cell via the AUCell package (version 1.24.0) (AUCell, RRID:SCR_021327) [31]. To account for the paired structure of our samples, we performed a statistical comparison using a linear mixed-effects model, which accommodates sample pairing while preserving the full resolution of single-cell data. We constructed two models: a null model containing only the random effect of paired samples and a full model that included the fixed effect of disease condition. A likelihood ratio test was then applied to compare these models, assessing the significance of the disease condition’s effect on NF-κB activity.

### 2.5. Differential NF-κB Target Regulation and Gene Ontology Analysis

To determine whether the NF-κB target genes regulated in RS relative to CLL are distinct, the gene set of high-confidence NF-κB targets obtained previously was used, and a differential gene expression analysis was performed between malignant cells from the two conditions. For this purpose, the FindMarkers function of the Seurat package (version 5.0.1) (Seurat, RRID:SCR_016341) was used in the malignant cells of CLL vs. RS and tested only genes detected in at least 10% of the cells in either condition [24]. Since the CLL condition was used as a reference for the comparison, genes with a log2 fold change (LFC) greater than 0.25 and adjusted *p* value ≤ 0.05 were considered differentially expressed targets (DETs) under the RS condition. DETs were used for overrepresentation analysis (ORA) through the clusterProfiler package (version 4.12.6) (clusterProfiler, RRID:SCR_016884) [32]. For this analysis, the significance limit was adjusted to *p* value ≤ 0.05. The gene sets used for the analysis were obtained from the Gene Ontology Biological Process (GOBP) database [33].

### 2.6. Differential Cell Composition Analysis

The relative cellular composition between the two studied conditions was compared. To achieve this, differences in the proportions of cell types were analyzed by a permutation test with the scProportionTest package (version 0.0.0.9), performing a total of 1000 permutations [34]. A cell group was considered to be significantly altered between conditions when it reached a false discovery rate (FDR) ≤ 0.05 with an LFC greater than 0.58.

### 2.7. Inference of Cell–Cell Communication Networks

To infer changes in cell signaling patterns, cell signaling networks were constructed separately for the CLL and RS samples via the CellChat package (version 2.1.2) (CellChat, RRID:SCR_021946) [35]. For this purpose, the CellChat ligand–receptor dataset, which includes the Secreted Signaling, ECM-Receptor, and Cell–Cell Contact categories, was used. The inferred communication probabilities at the signaling pathway level were then computed. Additionally, centrality scores were calculated for the networks, allowing the identification of dominant sender and receiver cell types for each ligand–receptor pair. Finally, the obtained networks were merged for a comparative analysis of the cellular communication networks between the two conditions [36].

### 2.8. Statistical Analysis

All the statistical analyses were performed using R software (version 4.4.1). *p* values of ≤0.05 were considered statistically significant. When appropriate, correction for multiple testing was applied via the Benjamini–Hochberg method, and an adjusted *p* value of ≤0.05 was considered statistically significant [37].

## 3. Results

### 3.1. Integration of scRNA-Seq Datasets and Comprehensive Cell-Type Annotation

**Figure 2:**
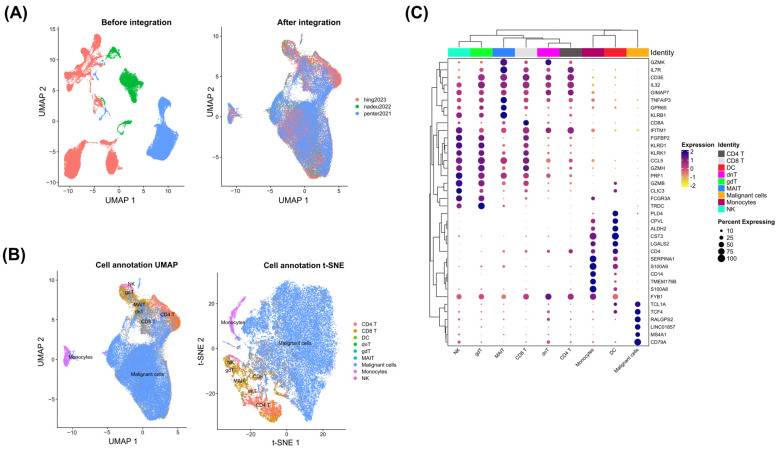
Dataset integration and cell-type annotation. (**A**) A dimensional reduction plot with UMAP reduction for the datasets. The left side of the graph shows the separation between the datasets before the integration process, whereas the right side shows it after the integration. The colors represent each study evaluated. (**B**) A dimensional reduction plot with both UMAP and t-SNE reduction for the cell-type annotation. The left side shows the cell-type groups in the UMAP reduction, whereas the right side shows these in the t-SNE reduction. The colors represent each cell type identified. (**C**) This dot plot shows the main markers found for each annotated cell type. The size of the dot represents the percentage of that cell group expressing the marker. The color of the dots represents the scale of the marker expression level. The color associated with the upper dendrogram represents the cell type.

To identify the activation state of NF-κB in the evolution of CLL to RS, we first collected scRNA-seq data from four patients with paired peripheral blood samples across three distinct studies. After data preprocessing and normalization, these data were integrated via the RPCA method (Figure 2A), resulting in a combined dataset of 49,879 cells. All subsequent analyses were based on this integrated dataset. Next, cell-type annotation was performed via a combination of clustering and reference-based methods. With this approach, a total of nine cell types were identified, namely, B cells (identified as malignant cells), monocytes, dendritic cells (DCs), natural killer (NK) cells, CD4+ T cells, CD8+ T cells, double-negative T cells (dnTs), gamma delta T cells (gdTs), and mucosal-associated invariant T (MAIT) cells (Figure 2B). All annotated cell types, except those labeled as malignant cells, were collectively referred to as immune cells in subsequent analyses. Additionally, the main gene expression markers of each cell type are shown in Figure 2C.

### 3.2. Key Components of the NF-κB Pathway Are More Widely Expressed in Malignant RS Cells

**Figure 3:**
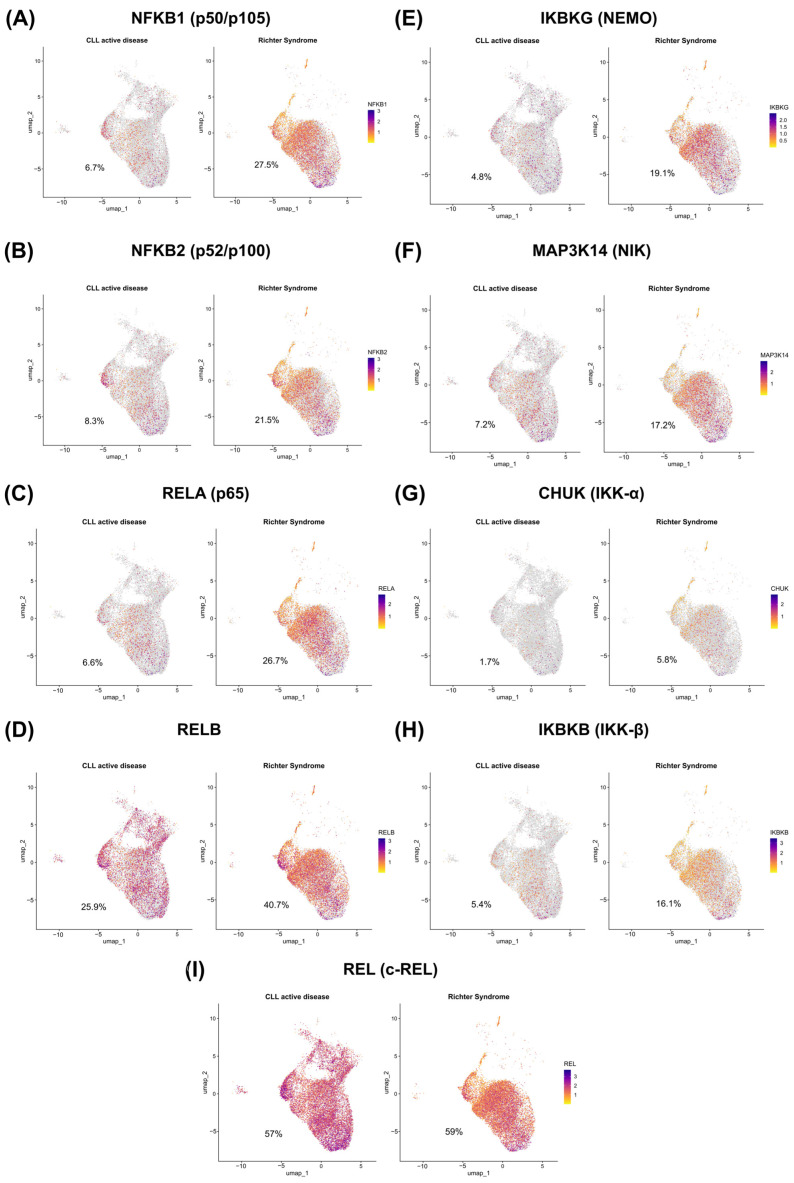
Evaluation of the expression of NF-κB pathway members in malignant CLL and RS cells. Overall, these dimensionality reduction plots demonstrate the expression level and percentage of malignant cells expressing NF-κB pathway components under each condition. The left side of each plot represents CLL, whereas the right side represents RS. The color gradient represents the expression level and is adapted for each target. (**A**) Expression level and percentage of malignant cells expressing *NFKB1* in CLL and RS. (**B**) Expression level and percentage of malignant cells expressing *NFKB2* in CLL and RS. (**C**) Expression level and percentage of malignant cells expressing *RELA* in CLL and RS. (**D**) Expression level and percentage of malignant cells expressing *RELB* in CLL and RS. (**E**) Expression level and percentage of malignant cells expressing *IKBKG* in CLL and RS. (**F**) Expression level and percentage of malignant cells expressing *MAP3K14* in CLL and RS. (**G**) Expression level and percentage of malignant cells expressing *CHUK* in CLL and RS. (**H**) Expression level and percentage of malignant cells expressing *IKBKB* in CLL and RS. (**I**) Expression level and percentage of malignant cells expressing *REL* in CLL and RS.

After identifying the cell types, only the malignant cells were filtered out initially. The percentage of these cells expressing NF-κB pathway components between the two conditions was compared. As a result, it was observed that the central members of the NF-κB pathway are more widely expressed in RS malignant cells than in CLL cells, mainly *NFKB1* (RS: 27.5% vs. CLL: 6.7%, Figure 3A), *NFKB2* (RS: 21.5% vs. CLL: 6.7%, Figure 3B), *RELA* (RS: 26.7% vs. CLL: 6.6%, Figure 3C), *RELB* (RS: 40.7% vs. CLL: 25.9%, Figure 3D), *IKBKG* (RS: 19.1% vs. CLL: 4.8%, Figure 3E), *MAP3K14* (RS: 17.2% vs. CLL: 7.2%, Figure 3F), *CHUK* (RS: 5.8% vs. CLL: 1.7%, Figure 3G), and *IKBKB* (RS: 16.1% vs. CLL: 5.4%, Figure 3H). The *REL* gene was the only component evaluated that had a comparable percentage of expression under both conditions (RS: 57% vs. CLL: 59%, Figure 3I).

Additionally, after differential gene expression analysis, the components *NFKB1* (LFC = 1.69), *NFKB2* (LFC = 0.81), *RELA* (LFC = 1.56), *IKBKG* (LFC = 1.36), *MAP3K14* (LFC = 0.87), *CHUK* (LFC = 1.01), and *IKBKB* (LFC = 0.87) were significantly more highly expressed in RS, whereas *REL* (LFC = −0.71) was more highly expressed in CLL. The *RELB* component (LFC = 0.03) did not demonstrate significant differential expression. These results are shown in Table 1. These findings highlight that not only do a greater proportion of malignant RS cells express key constituents of the NF-κB pathway, but these components are also expressed in greater quantities.

### 3.3. Malignant RS Cells Exhibit Increased NF-κB Pathway Activity

**Figure 4:**
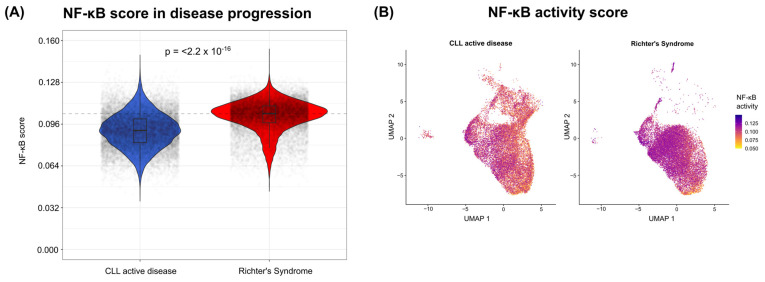
Evaluation of NF-κB activity between CLL and RS. (**A**) Comparison of the activity levels of the NF-κB pathway in CLL and RS patients. The dashed line represents the median activity of the RS group. The points presented in the graph represent the cells with jitter.. The blue color represents the CLL condition, and the red color represents the RS condition. NF-κB activity levels between conditions were compared using a linear mixed-effects model to account for paired samples. (**B**) The UMAP dimensionality reduction plot shows the distribution of NF-κB pathway activity levels among cells. On the left side is the representation of CLL, and on the right side the representation of the RS.

We then sought to verify whether the greater and broader expression of key components of the NF-κB pathway in malignant RS cells translates into a greater level of activity of this pathway. Focusing on the expression levels of NF-κB targets rather than the components themselves, a gene set of high-confidence NF-κB targets from CollecTRI was obtained, which contains a total of 1877 links. After filtering for unique targets present in the evaluated cells, a gene set comprising 976 genes was established. Using the AUCell method to calculate the NF-κB activity level in each cell on the basis of this gene set, a significantly greater (*p* < 0.001) level of NF-κB activity in malignant RS cells (Figure 4A) was observed. This pattern of activity was widely distributed among cells (Figure 4B). These results are consistent with the previous findings and indicate greater activity of the NF-κB pathway in malignant RS cells.

### 3.4. Differentially Regulated NF-κB Targets in Malignant RS Cells Are Associated with Key Processes of NF-κB Autoregulation, Cell Proliferation, and Immune Modulation

**Figure 5:**
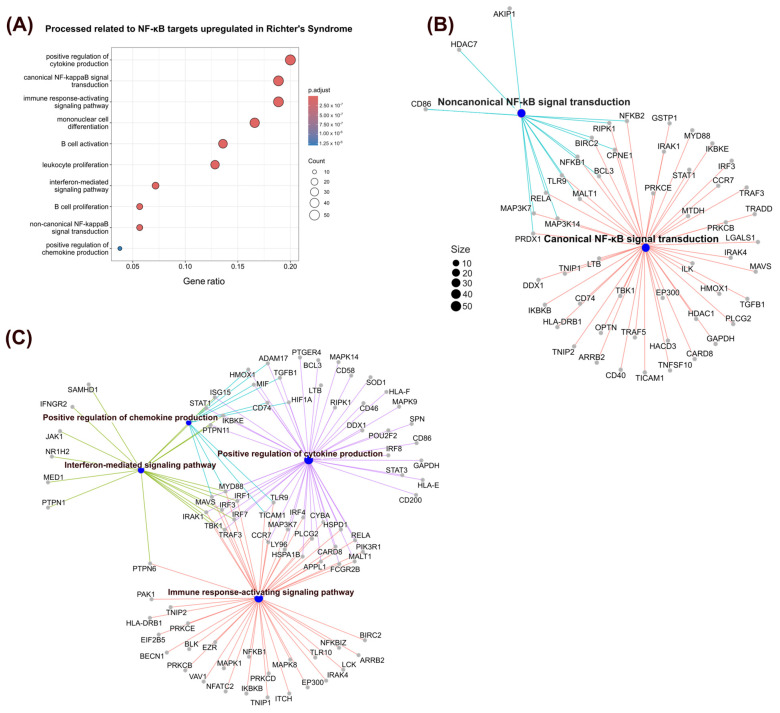
Biological processes enriched with NF-κB targets upregulated in RS. (**A**) The top 10 biological processes in which NF-κB targets were upregulated in RS are shown. The color gradient represents the adjusted *p* value, whereas the point size represents the number of targets found in the corresponding process. The gene ratio, on the x-axis, represents the fraction of upregulated targets found in the gene set. (**B**) A network representation of NF-κB targets found to be upregulated in RS that were associated with canonical NF-κB signal transduction (red lines) and noncanonical NF-κB signal transduction (light blue lines) processes. The blue dots represent the process, and the gray dots represent the associated genes. (**C**) A network of NF-κB targets associated with immune modulation processes, such as the immune response-activating signaling pathway (red lines), positive regulation of cytokine production (purple lines), the interferon-mediated signaling pathway (green lines), and positive regulation of chemokine production (light blue lines), was upregulated in RS. The blue dots represent the process, and the gray dots represent the associated genes.

To verify whether, in addition to the greater activity of the NF-κB pathway, the targets regulated by it are distinct in RS, the previously established gene set of NF-κB targets was used to conduct differential expression analysis. A total of 237 targets were upregulated in malignant RS cells compared with CLL cells (LFC ≥ 0.25 and adjusted *p* value ≤ 0.05). A list of all identified DETs is provided in Table S1. These targets were then used for ORA with GOBP gene sets. This analysis revealed a wide range of enriched processes for the evaluated DETs (Figure 5A), which included processes involved in NF-κB autoregulation (canonical and noncanonical NF-κB signal transduction), cell proliferation (B-cell activation and proliferation, and leukocyte proliferation), and immune modulation (cytokine and chemokine production and induction of mononuclear cell differentiation).

A deeper examination of these processes revealed that targets involved in both the canonical and noncanonical NF-κB pathways are upregulated in RS (Figure 5B). These targets include genes known to represent receptors involved in NF-κB activation, such as *TLR9* (LFC = 3.43; RS: 26.3% vs. CLL: 0.02%), *CD40* (LFC = 0.62; RS: 43.6% vs. CLL: 22.4%), and *MYD88* (LFC = 2.15; RS: 22.4% vs. CLL: 0.04%), in addition to the NF-κB components previously described (*NFKB1*, *NFKB2*, *RELA*, *MAP3K14*). Additionally, processes associated with immune modulation (Figure 5C), such as positive regulation of cytokine production, the immune response-activating signaling pathway, the interferon-mediated signaling pathway, and positive regulation of chemokine production, were also enriched among the DETs. These targets include genes known for these functions, such as *STAT3* (LFC = 1; RS: 52.9% vs. CLL: 22.8%) and *JAK1* (LFC = 0.32; RS: 70% vs. CLL: 44.9%). These findings of increased expression of receptors involved in NF-κB activation, in conjunction with immunological modulation, could indicate a role of the microenvironment in regulating greater NF-κB activity in malignant RS cells.

### 3.5. A Distinct Composition of the Immune Microenvironment Is Observed Between RS and CLL

**Figure 6:**
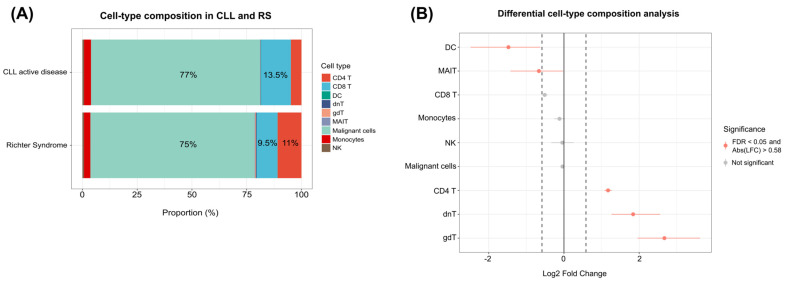
Cell-type composition in CLL and RS. (**A**) A proportion graph demonstrating the relative abundance of each cell type, represented by different colors. Owing to space limitations, only the percentage of the most abundant cells has been included in the text. (**B**) A dot plot representing the results of the differential cell-type composition analysis. The gray dots represent cells that had no difference in composition according to the established criteria (FDR < 0.05 and absolute log2 fold change > 0.58), whereas the red dots represent the cells that had a difference. The dashed line represents the log2 fold change values of −0.58 and 0.58. DC: dendritic cells; MAIT: mucosal-associated invariant T cells; CD8 T: CD8+ T cells; NK: natural killer cells; CD4 T: CD4+ T cells; dnT: double-negative T cells; gdT: gamma delta T cells; LFC: log2 fold change; FDR: false discovery rate.

To verify whether the differential composition of the immune microenvironment could be related to the evolution of CLL to RS, a broader view beyond malignant cells was considered. After a permutation test, a significant relative increase in the percentages of CD4+ T cells (RS: 11% vs. CLL: 4.8%), dnT cells (RS: 0.4% vs. CLL: 0.1%), and gdT cells (RS: 0.4% vs. CLL: 0.05%) was detected. On the other hand, a reduction in the percentages of DCs (RS: 0.05% vs. CLL: 0.14%) and MAIT cells (RS: 0.11% vs. CLL: 0.17%) was observed. Figure 6A shows an overview of these proportions, and Figure 6B shows the results of the permutation test. These data indicate that the composition of the immune microenvironment changes during disease progression.

### 3.6. Differential Cell–Cell Communication Patterns Drive NF-κB Activation in Malignant RS Cells

**Figure 7:**
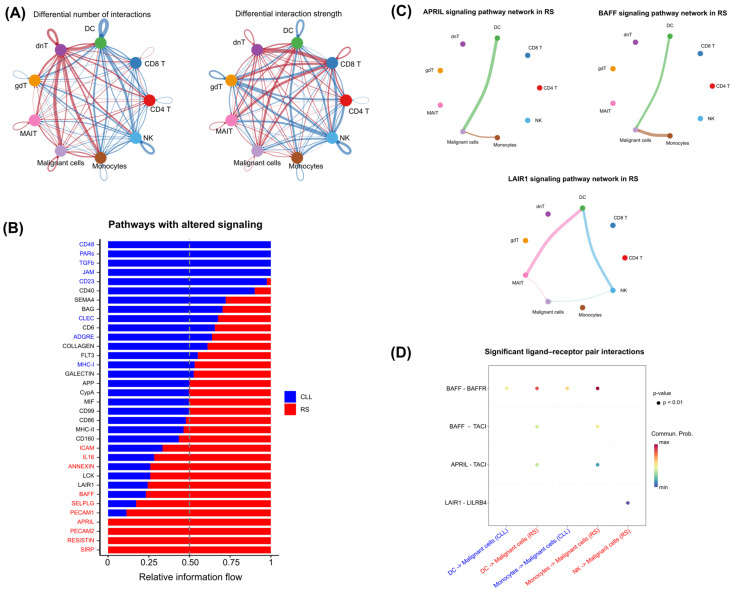
Changes in cell–cell communication patterns between CLL and RS. (**A**) A network representation demonstrating the differential number of interactions (left side) and differential interaction strength (right side) between different cell types across CLL and RS. The red line represents an increase in signaling in RS, whereas the blue line represents a decrease in signaling in RS. The arrows indicate the directionality of a cell’s signal relative to another cell or to itself. (**B**) The bar graph illustrates the relative information flow of each signaling pathway, which is calculated as the sum of the communication probabilities among all pairs of cell groups in the inferred network. In this graph, blue bars represent CLL, and red bars represent RS. Additionally, the pathways are ranked on the basis of the differences in information flow between the two conditions. A paired Wilcoxon test was performed to determine whether there was a significant difference in signaling information flow between the two conditions. Pathway names are colored to indicate significance: red indicates a significant difference favoring RS, whereas blue indicates a significant difference favoring CLL. (**C**) A network representation of the APRIL, BAFF, and LAIR1 signaling pathways in RS. The line represents signaling between two groups of cells, and the width of the line represents the intensity of this signaling. (**D**) Comparison of the communication probabilities of BAFF, APRIL, and LAIR1 signaling mediated by ligand–receptor pairs from cell groups to malignant cells. Only upregulated signaling by ligand–receptor pairs in RS has been demonstrated.

To verify whether, in addition to the change in the cellular composition of the immune microenvironment, the signaling patterns of these cells to malignant cells also change, cell–cell signaling networks were inferred and then compared across conditions. As a result, it was found that cell–cell signaling patterns differ during disease progression. A greater number of differential interactions were observed from CD4+ T cells, monocytes, DCs, dnT, gdT, and MAIT cells to malignant cells in RS (Figure 7A, left). Additionally, a greater differential interaction strength was observed from monocytes, DCs, dnTs, and gdTs to malignant cells in RS (Figure 7A, right). This finding indicates that even for cell types where no percentage differences were observed, a difference in the signaling pattern was found.

To understand which pathways were influenced by this differential signaling, the ligand–receptor interactions were analyzed at the pathway level and a significant increase in important pathways in RS, such as ICAM1, IL16, ANNEXIN, BAFF, SELPLG, PECAM1, APRIL, PECAM2, RESISTIN, and SIRP was found. In comparison, the pathways CD48, PARs, TGFβ, JAM, CD23, CLEC, ADGRE, and MHC-I were found to be significant in CLL. Figure 7B shows these results. Among these pathways, the pathways BAFF and APRIL, which are enriched in RS, have already been described as capable of activating the NF-κB pathway. Additionally, although it was not statistically distinct between conditions, the LAIR1 pathway—with 75% of the relative information flow associated with RS—has also been described as capable of activating the NF-κB pathway and was further examined.

Investigating the cell types involved in the signaling of these pathways, it was found that monocytes and DCs are the main cells that provide BAFF and APRIL signals to malignant RS cells, with NK and MAIT cells providing discrete signals in the LAIR1 pathway (Figure 7C). It was then found that the ligand–receptor pairs involved in this signaling pathway involve BAFF and APRIL ligands from DCs and monocytes that interact with the BAFF-R and TACI receptors on malignant RS cells, whereas NK cells signal through the LAIR1 ligand to the LILRB4 receptor (Figure 7D). These results align with previous findings and suggest that possible crosstalk involving the NF-κB pathway is active in malignant RS cells in response to the microenvironment, which is modulated as a consequence of NF-κB activation.

## 4. Discussion

Using an integrated dataset, a comprehensive analysis of the NF-κB pathway was performed during the progression of CLL to RS. With respect to the pathway components, not only higher expression levels, but also a broader expression pattern in RS for the main components of both the canonical (NFKB1 and RELA) and noncanonical (NFKB2 and RELB) pathways, as well as the downstream activators (IKBKG, MAP3K14, CHUK, and IKBKB), were found. It was subsequently demonstrated that this increased expression of the main components also translated into increased NF-κB pathway activity in malignant RS cells. This constitutive activation of NF-κB has been described as a frequent event in many hematologic malignancies, affecting both canonical and noncanonical pathways [38]. In CLL, elevated NF-κB pathway activity has been widely reported to be crucial for cell proliferation and survival [39]. In RS, initial studies have demonstrated genetic alterations in several components of the NF-κB pathway, including regulators, indicating the importance of this pathway in transformation [40,41,42]. Additionally, inhibition of the NF-κB pathway in patient-derived xenograft models of RS via IT-901, an NF-κB inhibitor that acts through c-Rel and p65, resulted in the induction of apoptosis and mitochondrial damage, highlighting its importance for malignant RS cell survival [43]. Similarly, previous work by Vaisitti et al. (2018), which established two patient-derived tumor xenograft models of RS, revealed overactivation of the NF-κB pathway, suggesting that this could be a characteristic of this condition [44]. Moreover, in the context of DLBCL, which represents the most common histological type of RS, the NF-κB pathway is known to be constitutively active and essential for the biology of the disease [45,46,47]. In these cases, activation of this pathway induces a dysregulation of cell survival and plays a critical role in sustaining the malignant phenotype [48,49,50]. Despite reported differences between de novo cases and CLL-derived DLBCL, these alterations could indicate a common and necessary mechanism in the aggressiveness of these lymphomas [41,51]. Although most of these studies did not directly assess the level of NF-κB activity or compare it with that in CLL, making it difficult to understand the dynamics of this activity during progression, they align with our results suggesting that the maintenance and modulation of NF-κB activity are key to RS biology.

The NF-κB pathway is known for its ability to regulate many targets depending on the signaling context and the dimers formed by its subunits [11,52]. The known NF-κB targets that are differentially regulated between CLL and RS and the functions in which they are involved were then analyzed. This analysis demonstrated that the differentially regulated NF-κB targets in RS are involved in processes such as NF-κB autoregulation, immune system modulation, and B-cell proliferation. Specifically, in the process of NF-κB autoregulation, genes encoding receptors associated with the activation of this pathway (both canonical and noncanonical), such as TLR9, CD40, and MYD88, were found to have greater and broader expression in malignant RS cells. CD40 has been demonstrated to be an important signaling complex for the activation of the NF-κB pathway in CLL [53,54]. The same has been described for TLR9, which also plays a role in the survival and proliferation of malignant cells in CLL [55,56]. The MYD88 molecule, a canonical adaptor for signaling via toll-like receptors (TLRs), including TLR9, is central to the activation of the NF-κB pathway through toll-like receptors [57]. Changes in MYD88 are associated with constitutive signaling through TLRs, contributing to the dysregulation of the NF-κB pathway and the development of an aggressive CLL phenotype [58,59]. A study by Fabbri et al. (2013) described genetic alterations in MYD88 in RS but highlighted that these alterations were already present at the CLL stage for the same patients, indicating that it is an early event in the progression of the disease [42]. Despite this, the difference in MYD88 expression observed in this analysis, which was approximately fourfold greater in malignant RS cells than in CLL cells, may indicate other mechanisms of transcriptional regulation of this molecule. The autoregulation of NF-κB is particularly relevant, as it creates a self-sustaining loop of pathway activation, which may be a distinguishing feature of RS compared with CLL. Therefore, these previous data combined with the current results could indicate an increase in alternative means of NF-κB activation in RS, driven by the NF-κB pathway itself in this specific context. This is particularly important given the description of reduced BCR signaling in RS, a central mechanism of NF-κB activation [21]. Additionally, recent studies demonstrate that both natural and synthetic inhibitors targeting MYD88 can effectively disrupt NF-κB signaling and exhibit anti-inflammatory and anti-cancer effects, which could indicate the importance of understanding its role in the progression of CLL to RS [60].

In addition to NF-κB autoregulation, NF-κB targets related to immune system modulation also represent important processes in RS biology. Modulation of the immune system is a widely described feature of CLL and is implicated in disease progression [13,61]. This process allows the exchange of signals between malignant CLL cells and a complex microenvironment that supports their growth and survival [61]. Thus, malignant CLL cells promote recruitment, activation, and constant modulation of their microenvironment [62]. Among the myriad of factors provided by the microenvironment in CLL, there are those that lead to the activation of NF-κB, such as BCR stimulation and signaling through TLRs [13]. In this sense, a study by Gould et al. (2021) demonstrated an increase in the expression of immune checkpoint molecules in the RS microenvironment [63]. Furthermore, immunomodulation is frequently described in RS, as it affects the expression of immunological regulators and the activation of pathways in malignant cells [64,65]. Although there is a limitation in directly comparing the composition of the microenvironment in the progression of CLL to that of RS, current results demonstrate a greater capacity for immune modulation by RS malignant cells. This modulation appears to occur through processes such as positive regulation of cytokine and chemokine production, as well as immune response pathways, as a consequence of activation of the NF-κB pathway. Given the data in the literature that indicate the importance of the microenvironment in RS, present data suggest that malignant cells begin to modulate their microenvironment through the production of factors that attract and modify the behavior of specific cells in the immune compartment. The consequences of modulating these cells for malignant RS cells remain to be explored.

After finding that NF-κB targets related to immune modulation are differentially regulated in RS, it was sought to verify the impact of this regulation on the microenvironment. Discrete changes were detected in some cell groups, such as an increase in CD4+ T cells, in RS samples compared with those in CLL samples. In addition, significant changes were found in the signaling patterns of cells in the microenvironment in RS samples, including those cell groups that had no numerical difference in composition. Interestingly, it was observed that these changes in signaling patterns affect pathways related to NF-κB activation, mainly BAFF and APRIL, with cells such as monocytes and DCs providing ligands for the receptors present on malignant RS cells. BAFF and APRIL are known activators of the NF-κB pathway [14,66]. In CLL, these two NF-κB activation pathways have been shown to be necessary for the proliferation and resistance to apoptosis of leukemic cells [67]. Additionally, the production of these factors is highly dependent on the microenvironment in CLL, which includes mainly nurse-like cells [68,69]. This finding contrasts with the results, where monocytes, which are capable of differentiating into nurse-like cells, were found to be the main source of BAFF and APRIL for malignant RS cells [68,70]. Taken together, these data seem to indicate a key role for NF-κB activation via BAFF and APRIL in RS. This may indicate an alternative mechanism by which NF-κB activation compensates for the decrease in BCR signaling in this context [21]. This phenomenon appears to be a consequence of complex and mutualistic crosstalk between RS malignant cells and the microenvironment, where NF-κB activation in RS malignant cells induces the production of microenvironmental modulators, which, in response, produce BAFF and APRIL, further activating NF-κB in malignant cells. This highlights not only the key role of NF-κB activation in the pathogenesis of RS but also the role of the modulation and adaptation of the microenvironment to provide the necessary signals for this activation.

There are several limitations in the current study, such as the small sample size and the use of peripheral blood samples. From these data, we cannot directly infer whether the observed pattern is reflected in the diseased tissue niches. However, the absence of paired CLL–RS samples from tissue niches makes such an approach difficult. Thus, additional studies are needed to investigate the differential activation status of the NF-κB pathway between CLL and RS in tissue niches (BM and SLO). Nevertheless, this is one of the first descriptions of the activation status of the NF-κB pathway in malignant cells during the progression from CLL to RS. Additionally, the mechanisms that regulate this activation were identified, contributing to the understanding of the dynamics of this pathway in this context.

## 5. Conclusions

In this study, an increase in NF-κB pathway activity was identified during the progression of CLL to RS, both at the level of component expression and pathway activity. This increase in activity was also reflected in the regulation of distinct targets under both conditions, with these targets involved in the autoregulation of the NF-κB pathway itself and in the modulation of the immune system. This modulation of the immune system was shown to result in a change in cell–cell signaling patterns, with cells in the microenvironment providing activation of the NF–κB pathway in malignant cells through alternative pathways to the BCR, such as BAFF and APRIL. Thus, the data indicate that the NF-κB pathway is key to the pathology of RS and that the microenvironment is modulated and adapted to provide the signals necessary for its activation. It is hoped that these results can contribute to the advancement of the current understanding of the dynamics of NF-κB action in the progression of CLL to RS and help to identify the main pathways for intervention in this disease progression.

## Figures and Tables

**Table 1:** Differential expression analysis of NF-κB components between CLL and RS.

Gene	Average Log2 Fold Change	% Expressed in RS	% Expressed in CLL	Mean Expression in RS *	Mean Expression in CLL *	Adjusted *p* Value
*NFKB1*	1.69	27.5	6.7	0.28	0.08	0 **
*NFKB2*	0.81	21.5	8.3	0.22	0.11	1.79 × 10^−251^
*RELA*	1.56	26.8	6.6	0.26	0.08	0 **
*RELB*	0.03	40.7	26	0.47	0.39	1.30 × 10^−85^
*REL*	−0.71	59	57.1	0.73	0.95	3.12 × 10^−142^
*IKBKG*	1.36	19.1	4.8	0.18	0.06	0 **
*MAP3K14*	0.87	17.2	7.3	0.18	0.09	1.32 × 10^−175^
*CHUK*	1.01	5.8	1.7	0.05	0.02	1.61 × 10^−92^
*IKBKB*	0.87	16.1	5.4	0.14	0.07	4.24 × 10^−228^

* Log-normalized mean expression levels. ** The adjusted *p* value is too low and therefore cannot be displayed.

## Data Availability

The raw data can be obtained from online databases including the GEO database (http://www.ncbi.nlm.nih.gov/geo, accessed on 3 September 2024) under the access numbers GSE165087 and GSE183432 and from the Zenodo repository (https://zenodo.org/, accessed on 6 September 2024) under the access number 6631966.

## Supplementary Materials

The following supporting information can be downloaded at: https://www.mdpi.com/article/10.3390/genes15111434/s1, Table S1: Differential expression of NF-κB targets between the malignant cells of RS and CLL.
